# Virtuous laughter: we should teach medical learners the art of humor

**DOI:** 10.1186/s13054-015-0927-4

**Published:** 2015-05-11

**Authors:** Simon Oczkowski

**Affiliations:** Hamilton General Hospital, McMaster Clinic, 4th floor, Room 434, 237 Barton St East, Hamilton, ON L8L 2X2 Canada

## Abstract

There is increasing recognition of the stress and burnout suffered by critical care workers. Physicians have a responsibility to teach learners the skills required not only to treat patients, but to cope with the demands of a stressful profession. Humor has been neglected as a strategy to help learners develop into virtuous and resilient physicians. Humor can be used to reduce stress, address fears, and to create effective health care teams. However, there are forms of humor which can be hurtful or discriminatory. In order to maximize the benefits of humor and to reduce its harms, we need to teach and model the effective and virtuous use of humor in the intensive care unit.

Medicine is a noble profession, and so physicians are expected to demonstrate virtuous character. In the hospital, we teach medical students and residents the knowledge, skills, and attitudes required to treat the critically ill. They learn how to comfort patients and their families during times of great suffering. Generally, we are good at teaching these skills. But increasingly, evidence shows that ICU workers have a high rate of burnout and job dissatisfaction [1,2]. We need to teach our learners not only how to care for the sick, but also how to care for themselves. We need to create novel methods for developing virtuous physicians, and at the same time we need to make the ICU a more humane environment for those who work there. And for that reason, I think it is time we begin to teach medical learners the art of humor.

It may at first seem morbid, but humor is one of the reasons I look forward to my work in critical care. Sharing laughs with patients, families, and staff is one of the most enjoyable parts of working in the hospital. Places traditionally associated with disease and death have always had close associations with humor. The very term used to describe this phenomenon, 'gallows humor', refers to one of the most horrific execution practices imaginable. Why do such dark places inspire jocularity? And how can teaching humor in the ICU promote the development of virtuous physicians?

Firstly, humor can relieve stress. In the ICU, where hours are long, tasks are intense, and emotions run high, healthcare providers struggle through their daily work routine with the constant threat of burnout. Laughter and humor are well-described coping methods [3]. By finding humor even in the darkest of places, we find a brief respite from what can sometimes be a draining place to work. Sharing laughter with others encourages camaraderie and the virtues of empathy and mutual respect.

Humor can also provide an outlet for our fears. Sometimes we see patients who remind us of our friends or families. Through them, we see our loved ones fall ill, and sometimes die. On occasion, we even have to take care of our loved ones, or valued members of our community. In the face of inevitable death, sometimes the only emotional outlet available are jokes. They can remind us of what is important by poking fun at those little things we take for granted. Patients also often make jokes when they are afraid. By finding humor even in the darkest of times, we remind ourselves of the joy in our lives, and find the strength to cope with our fears. In these situations, humor encourages the virtues of courage and perseverance.

Importantly, humor creates community. Sharing a laugh is a simple pleasure and a shared experience which brings people together [4]. In a place where life and death hang in the balance, it is easy to become self-important, arrogant, or closed-minded. By poking fun at ourselves, humor can keep us humble, and keep us together - working with, and for, the patients under our care. Humor can cultivate the virtues of humility and compassion.

But the use of humor in medicine has associated risks, as well as benefits. Jokes which poke fun at vulnerable patients, or which are discriminatory - laced with racism, sexism, or other cruel prejudices - are harmful. Just as humor can bring us together, it can be used to exclude and depersonalize others. It can deprive people of the respect they deserve. Inappropriate humor can do this whether these jokes are heard by patients and their families, or shared behind closed doors. Levity can also distract us from the seriousness of the situation. We are charged with the care of the critically ill, and we cannot be flippant in our delivery of the best possible care. For us, the ICU is the place where we live and work, day after day. For the patients and families, it is the place they dread coming to, night after night. We need to show understanding and respect for their plight. We need to demonstrate the virtues of temperance and self-reflection in our use of humor. We must avoid taking pleasure in the suffering of others.

## Conclusion

It is time we make a serious effort to teach medical students and residents the art of virtuous humor. Critics might say that curriculum space is too limited to dedicate time to jokes. Others may claim that developing virtues is outside the scope of the medical curriculum. I would argue, however, that such teaching need not be formal - we should teach virtuous humor by example. We can make jokes to keep our spirits up; to acknowledge our fears; and to create camaraderie. Just as importantly, we need to be role models for professionalism. Our jokes need to be rooted in ironic situations, clever word-play, and in our own flaws - jibes should not at the expense of the people we care for. We need to take the job seriously - but take ourselves in good humor.
